# Effects of Protocatechuic Acid (PCA) on Global Cerebral Ischemia-Induced Hippocampal Neuronal Death

**DOI:** 10.3390/ijms19051420

**Published:** 2018-05-09

**Authors:** A Ra Kho, Bo Young Choi, Song Hee Lee, Dae Ki Hong, Sang Hwon Lee, Jeong Hyun Jeong, Kyoung-Ha Park, Hong Ki Song, Hui Chul Choi, Sang Won Suh

**Affiliations:** 1 Department of Physiology, College of Medicine, Hallym University, Chuncheon 24252, Korea; rnlduadkfk136@hallym.ac.kr (A.R.K.); bychoi@hallym.ac.kr (B.Y.C.); sshlee@hallym.ac.kr (S.H.L.); zxnm01220@gmail.com (D.K.H.); bluesea3616@naver.com (S.H.L.); 2 Department of Medical Science, College of Medicine, Hallym University, Chuncheon 24252, Korea; jd1422@hanmail.net; 3 Division of Cardiovascular Disease, Hallym University Medical Center, Anyang 14068, Korea; pkhmd@naver.com; 4 College of Medicine, Neurology, Hallym University, Chuncheon 24252, Korea; hksong0@hanmail.net (H.K.S.); dohchi@naver.com (H.C.C.)

**Keywords:** global ischemia, neuron death, protocatechuic acid, oxidative stress, blood–brain barrier, microglial activation

## Abstract

Global cerebral ischemia (GCI) is one of the main causes of hippocampal neuronal death. Ischemic damage can be rescued by early blood reperfusion. However, under some circumstances reperfusion itself can trigger a cell death process that is initiated by the reintroduction of blood, followed by the production of superoxide, a blood–brain barrier (BBB) disruption and microglial activation. Protocatechuic acid (PCA) is a major metabolite of the antioxidant polyphenols, which have been discovered in green tea. PCA has been shown to have antioxidant effects on healthy cells and anti-proliferative effects on tumor cells. To test whether PCA can prevent ischemia-induced hippocampal neuronal death, rats were injected with PCA (30 mg/kg/day) per oral (p.o) for one week after global ischemia. To evaluate degenerating neurons, oxidative stress, microglial activation and BBB disruption, we performed Fluoro-Jade B (FJB), 4-hydroxynonenal (4HNE), CD11b, GFAP and IgG staining. In the present study, we found that PCA significantly decreased degenerating neuronal cell death, oxidative stress, microglial activation, astrocyte activation and BBB disruption compared with the vehicle-treated group after ischemia. In addition, an ischemia-induced reduction in glutathione (GSH) concentration in hippocampal neurons was recovered by PCA administration. Therefore, the administration of PCA may be further investigated as a promising tool for decreasing hippocampal neuronal death after global cerebral ischemia.

## 1. Introduction

Cerebrovascular disorders encompass a diverse range of neurological diseases, such as stroke, myocardial infarction, vascular dementia, and chronic cerebral hypoperfusion [1]. Particularly, ischemic stroke causes one of the most severe cerebropathologic conditions, accounts for 88% of all stroke patients, and represents various clinical signs of focal or global cerebral dysfunction [2]. While focal ischemia can cause local damage and identify infarct volume, the advantage of global cerebral ischemia is that we can confirm the selective and delayed neuronal cell death of, especially, the cornu ammonis 1 (CA1) region in the hippocampus [3]. Global cerebral ischemia (GCI) accompanies the degeneration of the neural tissues, leading to hippocampal neuron death and cognitive deficits. Ischemia-induced brain damage can be recovered via early reperfusion, but this reperfusion can, itself, become an initiation mechanism of a cell death pathway that is caused by blood reperfusion, blood–brain barrier (BBB) disruption, microglia activation and zinc release. Furthermore, ischemia-reperfusion injury is due to free radical production at the onset of blood reintroduction after global cerebral ischemia [4]. Superoxide and other reactive oxygen species produced by the ischemic insult and zinc release can lead to the production of oxidative stress. Reactive oxygen species (ROS) reverses the protein-mediated sequestration of zinc and thus increases the intracellular free zinc levels, which increases activation of ROS formation. If it becomes elevated for sustained periods, it can cause neuronal death. The brain is especially susceptible to oxidative stress because neurons have high levels of polyunsaturated fatty acids and, on the other hand, the concentration of endogenous antioxidant enzymes found in neuronal tissues are too low to buffer against this increased ROS activity. Therefore, oxidative stress may lead to hippocampal neuronal cell death owing to ischemia and subsequent blood reperfusion.

Protocatechuic acid (PCA), a type of the major benzoic acid or phenols, a derivative from vegetables, fruits, and many Chinese herbal medicines, is a primary metabolite of antioxidant substance. Some studies with animal models have stated that PCA shows antitumor promotion effects and strong anti-oxidant behavior [5,6,7]. It has been suggested that PCA may be regarded as a therapeutic candidate for the administration of neurodegenerative diseases, such as Parkinson’s disease [8]. The mechanism is mainly related with antioxidant activity, including prevention of free radical production, as well as removing free radicals and up-regulation of enzymes that are involved in their neutralization. A recent in vivo study demonstrated that PCA protected hepatic cells from hepatic injury caused by tertbutyl hydroperoxide [9,10]. Therefore, this experimental evidence has confirmed that phenolic compounds may offer several biological effects, such as anti-inflammatory, antibacterial, antioxidant, anti-diabetic, and neuroprotective properties [11,12].

Although information about PCA’s protective property in tissue and organs has been widely reported [13], the effect of PCA on global cerebral ischemia-induced hippocampal neuronal death has not been studied. Thus, the present study investigates the possible neuroprotective effects of PCA on GCI-induced hippocampal neuron death.

## 2. Results

### 2.1. PCA Reduces Neuronal Death after Global Ischemia

**Figure 1:**
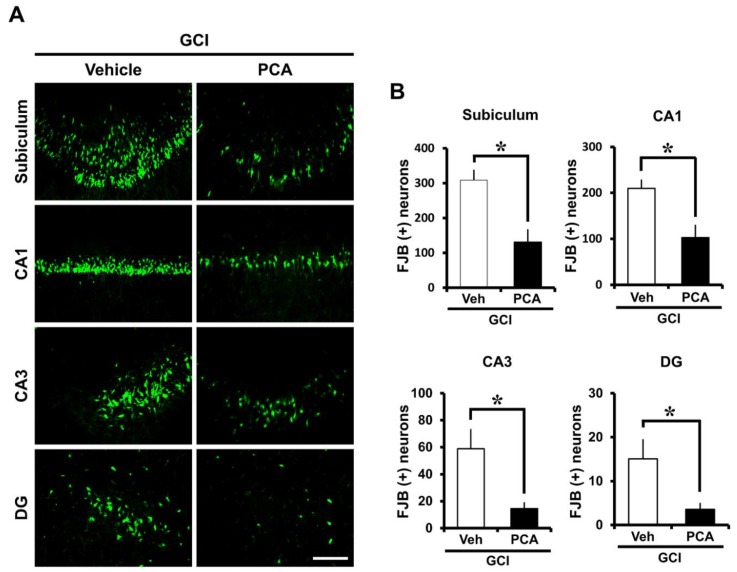
PCA treatment reduces ischemia-induced hippocampal neuronal death. Global ischemia induced neuronal death in the hippocampal areas: subiculum (Sub), CA1, CA3, and dentate gyrus (DG) one week after ischemic insult. Figure (**A**) shows FJB (+) neurons in the CA1, CA3, subiculum and DG. Oral post-treatment of PCA (30 mg/kg) for one week observed neuroprotective effect in the CA1, CA3, subiculum, and DG after ischemia (*n* = 9). Scale bar = 100 μm. (**B**) Bar graph indicating the quantification of degenerating neurons in the hippocampus. The number of FJB (+) neurons is reduced in PCA (30 mg/kg)-treated group in the subiculum, CA1, CA3, and DG compared to the vehicle-injected group. Data are mean ± S.E.M., *n* = 5 from each sham group. *n* = 9 from each ischemia group. * Significantly different from the vehicle-treated group, *p* < 0.05.

**Figure 2:**
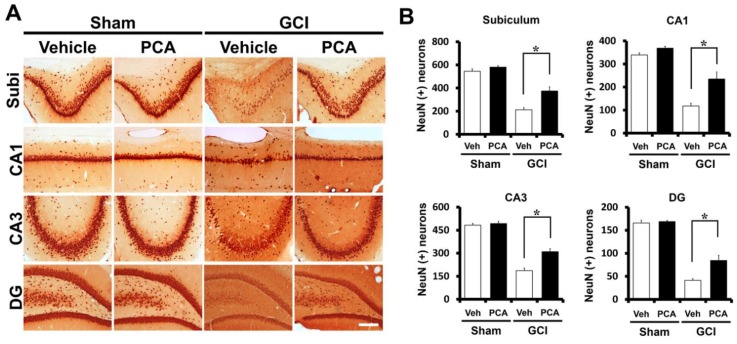
PCA treatment promotes neuronal survival following ischemia. Ischemia-induced neuronal loss was measured by NeuN staining at one week after global ischemia or sham operation. Figure (**A**) representative photomicrographs show significant neuronal loss in the CA1, CA3, subiculum, and DG after ischemia (*n* = 9). However, ischemia-induced NeuN (+) neurons were preserved by PCA treatment in the hippocampal regions compared to vehicle treatment. Scale bar = 100 μm. (**B**) This graph represents the quantitated live neurons (NeuN (+) neuron) in the hippocampus. Data reflect the mean ± S.E.M., *n* = 5 from each sham group. *n* = 9 from each ischemia group. * Significantly different from the vehicle-treated group, *p* < 0.05.

Severe neuronal death is produced at seven days after ischemia. To investigate whether PCA has neuroprotective effects after ischemia, the rats were sacrificed at 1 week after ischemia with or without PCA treatment. After insult, we conducted NeuN staining in order to detect surviving neurons, and also FJB (Fluoro-Jade B) staining in order to detect degenerating neurons in the hippocampal subiculum (Sub), the cornu ammonis 1 (CA1), the cornu ammonis 3 (CA3) and dentate gyrus (DG) area. Firstly, FJB staining showed broad neuronal death in the subiculum (sub), CA1, CA3, and dentate gyrus (DG) of the hippocampus (Figure 1) (*p* < 0.05). This staining provides a selective marker of degenerating neurons. No degenerating neurons were present in the sham-operated brain sections. The number of degenerating neurons between the sham and ischemia-induced groups was highly contrasting. Compared to the vehicle-treated group, rats given PCA (30 mg/kg, p.o) after ischemia displayed a remarkable low number of degenerating neurons in the hippocampus (Figure 1A). As depicted in Figure 1B, PCA-treated group had 51%, 75%, and 76% fewer degenerating neurons in the CA1, CA3, and DG, and 58% in the subiculum than in the vehicle-treated group, respectively (Figure 1B). Moreover, NeuN staining confirmed that PCA treatment showed an increased number of live neurons compared to the vehicle-treated group, 43%, 50%, 40%, and 51% more live neurons in subiculum, CA1, CA3, and dentate gyrus than the vehicle-treated group (Figure 2A,B) (*p* < 0.05). These results indicate that PCA treatment shows neuroprotective effects after transient cerebral ischemia.

### 2.2. PCA Reduces Ischemia-Induced Oxidative Injury

**Figure 3:**
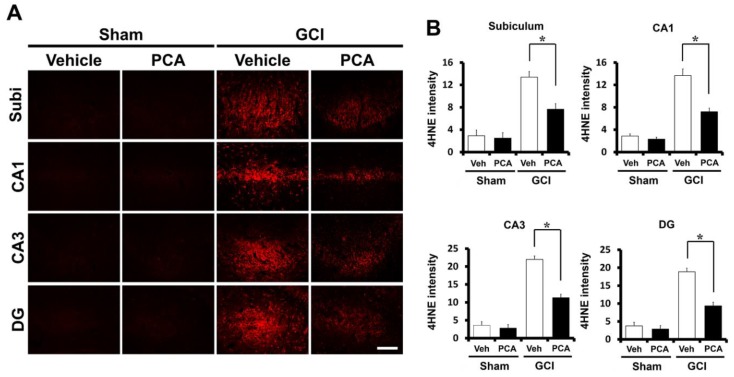
PCA treatment reduces oxidative damage after ischemia. Neuronal oxidative damage was detected by 4HNE (red color) staining in the hippocampal CA1, CA3, DG (dentate gyrus) and subiculum area at seven days following ischemia. As shown in Figure 2, PCA treatment alleviated damage by oxidative stress in the CA1, CA3, DG, and subiculum after insult. (**A**) Sham-operated groups display minimal 4HNE staining in the hippocampus. Oral injection of PCA for 1 week reduced the intensity of the 4HNE fluorescence compared to the saline-treated group after ischemia. Scale bar indicates 100 µm. (**B**) The bar graph shows the 4HNE fluorescence intensity in the CA1, CA3, DG, and subiculum. The fluorescence intensity reflects a significant distinction between saline- and PCA-treated groups. Data reflect the mean ± S.E.M., *n* = 5 from each sham group. *n* = 9 from each global ischemia group. * Significantly different from the vehicle-treated group, *p* < 0.05.

To determine the degree of oxidative damage after ischemia, we evaluated oxidative stress via 4-hydroxynonenal (4HNE) staining. Brain samples were immunohistochemically stained with a 4HNE antibody at one week after global cerebral ischemia induction to find whether oxidative stress had occurred in hippocampal neurons. In case of the sham-operated group, saline or PCA-injected sections had no difference in the intensity of 4HNE staining in the hippocampus. 4HNE intensity was increased in the hippocampus of ischemia-induced rats due to insult. However, 4HNE intensity was reduced in the PCA-treated rats after ischemia (Figure 3A) (*p* < 0.05). As demonstrated in Figure 3B, the group treated with PCA had around 47% and 49% reductions in the intensity of 4HNE staining in CA1 and CA3, 50% in the DG, and 43% in the subiculum than the vehicle-treated group (Figure 3B).

### 2.3. PCA Prevents Ischemia-Induced Blood–Brain Barrier (BBB) Disruption

**Figure 4:**
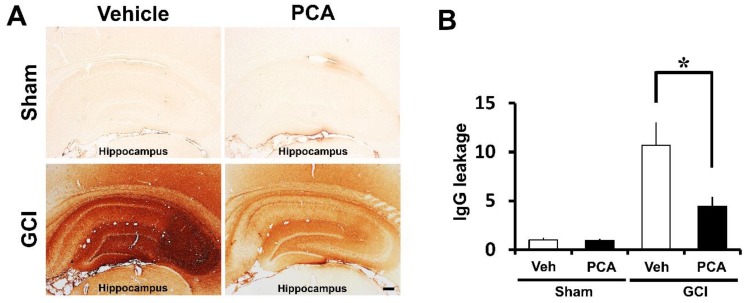
PCA administration reduces BBB disruption after ischemia. Images display a leakage of immunoglobulin through the blood–brain barrier (BBB) disruption in the hippocampus after global ischemia. Figure (**A**) shows low magnification (4×) photomicrographs of IgG staining in the hippocampus. At one week after ischemia, the entire hippocampus showed a high degree of immunoglobulin leakage, suggesting that serious BBB breakdown has occurred in ischemia-induced rats (*n* = 9). PCA (30 mg/kg)-injected rats (*n* = 9) for 1 weeks after ischemia decreases the IgG leakage in the whole hippocampus compared to the ischemia vehicle group (*n* = 9). Scale bar = 200 μm. (**B**) The bar graph shows the degree of IgG leakage in the whole hippocampus. The degree of IgG extravasation was reduced in the PCA (30 mg/kg)-treated group. Data reflect the mean ± S.E.M., *p* < 0.05.

To evaluate the degree of blood–brain barrier disruption, we stained brain sections to detect extravasation of serum IgG by using immunohistochemistry as described previously [14,15]. In sham-operated brain sections, leakage of IgG was shown to be minimal. However, in ischemia-induced rats, we observed significant extravascular IgG leakage in the hippocampus. Leaked IgG made coronas with a concentration gradient around vessels in the hippocampus region, infiltration of IgG in the hippocampus was seen across broad areas in whole brain sections. Ischemia-induced rats had around 90% more extravasation of serum IgG compared to sham-operated rats in the hippocampus. However, PCA-treated brain sections showed 58% lower immunoglobulin leakage compared to vehicle-treated group in the hippocampus after ischemic injury. Based on the results of the above IgG leakage from the hippocampus, it showed great dissimilarity between the two groups, ischemia-induced rat with saline, and with PCA (Figure 4) (*p* < 0.05).

### 2.4. PCA Decreases Ischemia-Induced Inflammatory Responses Mediated by Microglia and Astrocytes

**Figure 5:**
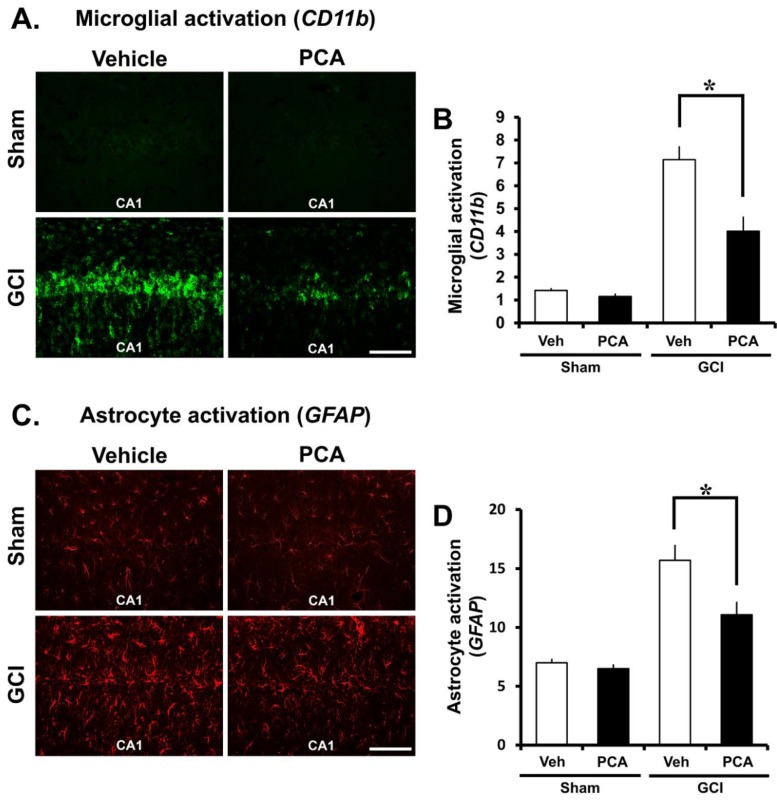
PCA treatment reduces microglia and astrocyte activation. Global ischemia induces an inflammatory response by promoting microglia activation and activated astrocytes, in the damaged region. In this figure, PCA treatment for one week prevents microglia and astrocyte activation in CA1 following global ischemia. (**A**) shows microglia activation and (**C**) shows astrocyte activation in the CA1 of hippocampus from sham-operated or ischemia-induced rats. It was increased in the ischemia-induced group in comparison to the sham-operated group. However, PCA administration prevented microglia and astrocyte activation in the PCA-treated groups after ischemia. Scale bar = 100 µm. (**B**,**D**) The bar graph represents the grade of microglia activation and the intensity of activated astrocytes in the CA1 region. Data reflect the mean ± S.E.M., *n* = 5 from each sham group. *n* = 9 from each ischemia group. *p* < 0.05.

Global ischemia is usually followed by inflammation induced by microglia and astrocytes. It is mentioned that inflammation contributes to ischemia injury [16,17]. The effect of the PCA treatment on activation of microglia and astrocyte after ischemia were detected by using the surface marker CD11b and GFAP at seven days after insult. We conducted assessment of microglial morphology, number, and intensity and intensity of astrocytes in the four groups (Sham (Vehicle, PCA) and ischemia (Vehicle, PCA)). Sham-operated groups had resting microglia and small astrocyte. Global cerebral ischemia triggered microglial activation, which reflecting macrophage activity. Also, activated astrocytes in ischemia are potentially harmful because they can express NOS and produce the neurotoxic NO [18]. In the present study, we discovered that microglial and astrocytes activation in CA1 region were rose around 64% and 43% respectively in the ischemia group in comparison to the sham-operated group. Microglial activation and reactive astrocytes were reduced around 35% and 23% in the PCA-treated group compared to the saline-treated group following ischemia. Therefore, this result demonstrates that PCA reduces microglial activation and reactive astrocytes after global cerebral ischemia (Figure 5A–D) (*p* < 0.05).

### 2.5. PCA Reverses Ischemia-Induced Glutathione (GSH) Deprivation

**Figure 6:**
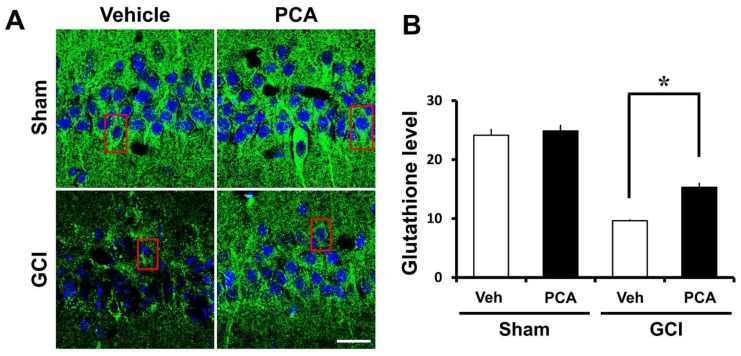
PCA administration rescues glutathione depletion after ischemia. This image displays gaps in glutathione (GSH) levels. (**A**) The sham-operated group include a mostly plentiful glutathione grade in the hippocampus. This image indicates GS-NEM (+) neurons of four groups; sham-vehicle (*n* = 3), sham-PCA (*n* = 3), ischemia-vehicle (*n* = 3), and ischemia-PCA (*n* = 3). Oral injection of PCA for one week after global ischemia improved GS-NEM fluorescent intensity in the CA1 region more than the saline-treated group. Scale bar = 20 μm. (**B**) The bar graph shows that the evaluation of GS-NEM fluorescence intensity in the CA1 region of four groups. The fluorescence intensity is significantly different between the vehicle and PCA-treated group. Data reflect the mean ± S.E.M., *n* = 3 from each sham group and the ischemia group. * Significantly different from the vehicle-treated group, *p* < 0.05.

Glutathione, an endogenous antioxidant, is important in controlling the redox response under the physiological environment of the human body. Brain damage owing to ischemia, seizure, and hypoglycemia reduce glutathione levels and then disrupt the overall redox system [19]. Thus, to investigate the effects of PCA on GSH reduction in a global ischemia setting, we performed GE-NEM staining with brain samples (30 µM) (Figure 6A) (*p* < 0.05). Then, we detected the GSH levels in the hippocampal CA1 region (Figure 6B). As a result, GSH levels were decreased in the CA1 at seven days following ischemia. However, GSH levels in the same region was increased around 37% thanks to PCA treatment compared to the saline-treated group in global cerebral ischemia.

### 2.6. PCA Reduces Intracellular Free Zinc Level

**Figure 7:**
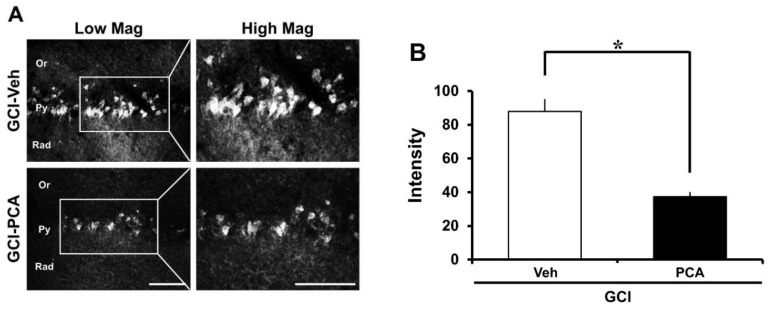
PCA administration decreases the intracellular free zinc level in the CA1 region. Ischemic neuronal death is promoted by zinc release and accumulation. However, oral treatment with PCA for 3 days reduces the intracellular free zinc level. (**A**) displays photomicrographs of TSQ fluorescence staining in CA1. After insult, the intracellular free zinc levels were increased. However, we confirmed that zinc levels were significantly decreased in the group that was administered PCA after ischemia. Scale bar = 100 μm (**B**) the bar graph indicates the TSQ fluorescence intensity from CA1. Data are mean ± S.E.M., *n* = 4 from each global cerebral ischemia group and ischemia group with PCA. * Significantly different from the vehicle-treated group, *p *< 0.05.

Global cerebral ischemia induces zinc release in CA1. This zinc release is known to promote both neuronal NADPH oxidase activity, which is the primary source of ROS responsible for the neuronal cell death seen in this setting. Therefore, intracellular zinc levels need to be tightly controlled. Under physiological conditions, cellular zinc levels are regulated by zinc transporters, zinc binding proteins and zinc sensors [20]. However, under conditions such as ischemia/reperfusion injury, traumatic brain injury or seizure, zinc homeostasis is destroyed and thus neuronal death occurs. Therefore, we performed TSQ fluorescence staining to reveal whether PCA can decrease intracellular zinc level in hippocampal CA1. As quantified in Figure 7, the intensity of TSQ staining within the intracellular CA1 region was decreased in the PCA-treated group compared to the saline-treated group after ischemia (Figure 7A,B) (*p* < 0.05).

### 2.7. PCA Improves Cognitive Function after Ischemia

**Figure 8:**
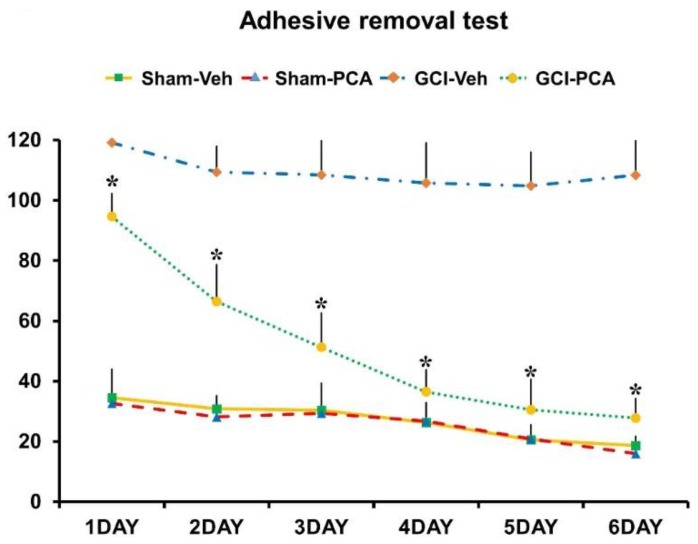
PCA administration improves cognitive function after ischemia. Rats administered PCA for 7 days after ischemia were evaluated for changes in cognitive and sensory function by the adhesive removal test. The graphs show the measured cognitive ability from each group. As a result, injection of PCA lead to increased improvement of ischemia-induced cognitive impairment, compared to the vehicle-treated group. Data reflect the mean ± S.E.M., *n* = 5 from each sham group and *n* = 8 form each ischemia group. * Significantly different from the vehicle-treated group, *p *< 0.05.

To test whether oral treatment of PCA showed improvement of cognitive function after ischemia, we analyzed behavior using a standard adhesive removal test protocol. The adhesive removal test (also referred to as the tape removal test) has commonly been used as a measure of motor co-ordination and sensory neglect after ischemia in experimental research using rats and mice [21,22,23,24,25,26]. We conducted this test for six days, starting the next day after surgery. As a result, the ischemia-induced group failed to get rid of the tape for maximum 120 s. However, the PCA treated group showed improved performance and removed the tape faster than in the vehicle-treated group after ischemia (Figure 8).

## 3. Discussion

The present study investigated the potential therapeutic effects of protocatechuic acid (PCA), one of the major phenol derivatives, on global cerebral ischemia-induced neuronal death. So far, we found that PCA significantly reduced oxidative stress, blood–brain barrier breakdown, activation of microglia and astrocytes, and neuronal death. These results suggest that reduction of oxidative stress by PCA may be used as a new therapeutic tool for preventing ischemic neuronal death.

The molecular mechanisms of global cerebral ischemia-induced neuronal death are highly complex. Although several laboratories have extensively investigated these mechanisms over the decades the precise mechanisms of ischemic neuronal death are still not clear. Previous studies have demonstrated that neuronal death was initiated during blood reperfusion period after certain period of ischemic state [27,28]. This ischemic-reperfusion can cause blood–brain barrier (BBB) breakdown and subsequently promote leukocyte infiltration and brain inflammatory process activation. Our previous study demonstrated that superoxide production by NADPH oxidase activation occurred during the blood reperfusion period after ischemia, which led to a series of neuronal death processes [27].

The present study was performed to prove the question of whether administration of PCA, known as an antioxidant, can prevent ischemia-induced hippocampal neuronal death. Muley et al. showed that a high concentration of PCA (200 mg/kg) reduced infarct volume, brain edema, and reactive oxygen species production [29]. However, they did not show individual hippocampal neuronal death, microglia activation, BBB disruption, and lipid peroxidation, as shown in our present study. In the present study we used a low dose of PCA (30 mg/kg) to see if this chemical has any neuroprotective effects after global cerebral ischemia. Kakkar et al. showed that a high dose of PCA induced GSH depletion and liver toxicity. They suggested that 100 mg/kg of PCA showed no toxicity [5]. Since PCA shows poor absorption by intestine, less than 100 mg/kg of PCA is nontoxic and a relatively safe. Therefore, we selected a dose of 30 mg/kg of PCA for use in this study to investigate whether oral administration has any neuroprotective effect on global ischemia-induced hippocampal neuronal death. Our previous studies have also demonstrated that a low dose of PCA (30 mg/kg) has neuroprotective effects after seizure or after traumatic brain injury [30,31]. We hypothesized that PCA may prevent BBB disruption, microglial activation, and neuronal death through reduction of ROS production by regeneration of glutathione contents. PCA is unusually effective in inhibiting neuronal cell death through oxidative stress in cultured PC12 cells [32]. Thus, PCA decreased neuronal death and prevented cognitive impairment by decreased oxidative stress [33]. PCA also showed protective effects on blood-brain barrier disruption, leading to neuroprotection [34]. With these previous studies, we hypothesized that administration of PCA can prevent ischemia-induced hippocampal neuronal death by scavenging or decreasing the production of ROS.

In the present study, we detected that PCA significantly lessened ischemia-induced neuronal death. The consequence of FJB staining demonstrated a marked decline of number of degenerating neurons in the hippocampal subiculum, CA1, CA3, and dentate granule cells in the PCA-administrated group compared to the vehicle-administered group after ischemia. This trend correlated with the NeuN staining results, which represented an increased number of live neurons in the PCA group. The consequences of FJB and NeuN staining reflected neuroprotective effects of PCA after global cerebral ischemia.

Ischemia/reperfusion produces a burst of free radical production, which eventually drives oxidative stress and subsequent brain injury. This formation of ROS occurs because of (i) iron-associated free radical formation; (ii) depletion of antioxidant enzymes; (iii) increase of lipids and fatty acids; and (iv) production of brain edema [35]. PCA, extracted from green tea, shows protective effects on H_2_O_2_-induced apoptosis and oxidative stress through increased glutathione (GSH) levels in cultured PC12 cells [36]. One study concluded that the neuroprotective mechanism of PCA was via functioning as endogenous antioxidants, where PCA promoted cell growth by quenching H_2_O_2_ [37]. They concluded that PCA can exert protective effects against tissue damage caused from superoxide formation and ROS-dependent stress [38]. Another study stated that the reduction of GSH levels in metal-treated animals suggests that a metal potentially binds to the ROS-related enzyme active site or interacts with the active amino acids of this enzyme and, thus, promotes the accumulation of free radicals [39]. Thus, they observed improvement in antioxidant enzymatic activity owing to PCA treatment and concluded that PCA can prevent production of reactive oxygen species and inhibit expansion of tissue injury [6]. Given this, PCA may act as a therapeutic tool for brain recovery and, thus, may be responsible for neurodegenerative disease repair [40]. The blood-brain barrier (BBB) balances nutrients and ions at necessary levels for proper neuronal function, and takes the neurotoxic substrates away from the brain. After global ischemia insult, the BBB can be disrupted, leading to plasma components, such as leukocytes and erythrocytes, crossing the BBB. This BBB disruption gradually increases intracerebral hemorrhage, neurodegenerative processes, inflammation, trauma, and vascular disruption. This process generates neurotoxic substrates that damage synaptic and neural transmission and brain function [41,42]. Furthermore, brain ischemia further breaks the BBB permeability and severely worsens brain edema, which leads to the development of brain penumbra and neurological disorder [43]. Therefore, BBB protection is a principal aim in alleviating brain edema and inhibiting cerebral ischemia. Additionally, brain injury induced by ischemia, hypoglycemia, and seizure leads to more severe neuroinflammation [44,45]. The inflammatory markers, such as microglia, ROS, IL-1β, and TNF-α, were detected in patents who experienced brain injuries. These inflammatory factors may penetrate the BBB and then enter intracerebral circulation. These factors may promote activation of peripheral immune responses [46], causing disruption of the blood-brain barrier and then serum IgG release from the cerebral blood vessels. Therefore, to evaluate the effects of PCA on BBB disruption, we performed IgG staining on brain sections. In the present study, we found that IgG-stained intensity as a function of area was reduced in the PCA-administrated group compared to the vehicle-treated group after ischemia. This result suggests that neuroprotection by PCA administration might be associated with recovery or protection from BBB disruption, which is one of the central mechanisms associated with ischemic neuronal injury [47]. PCA displayed protective effects against early ischemic BBB disruption via regulation of tight junctions (TJs) and the protein kinase C-alpha (PKCα) signal pathway. Furthermore, PCA has the ability to prevent BBB damage in ischemia-reperfusion injury by controlling MMP-9, which is a key contributing factor of BBB disruption and TJ proteins. PCA decreased oxidative stress-induced BBB damage through reduction of oxidative stress [48,49].

Astrocytes and microglia play an important role in hypoxemia, inflammation, and neurodegenerative diseases, such as ischemia, in the central nervous system (CNS). Under ischemic conditions, astrocytes and microglia can be rapidly activated by neurotoxic compounds or pro-inflammatory factors, such as TNF-α and IL-1β, which cause neuroinflammation [50]. A previous study demonstrated that regulation of astrocyte activation can reduce cytokine formation and thus protect neurons from ischemic injury [51]. Therefore, brain inflammation has been regarded as a potential target to treat stroke for several years, and a wide variety of approaches have been conducted to suppress ischemia-induced brain inflammation. The CA1 region of the hippocampus is vulnerable to microglial activation and reactive astrocytes in our global ischemia model. Thus, the present study evaluated microglial activation by CD11b and astrocyte activation by GFAP immunofluorescent staining. In the present study, we found that PCA treatment reduced microglial activation and reactive astrocytes after ischemia. This result suggests that PCA can prevent inflammatory processes after stroke.

Glutathione (GSH), composed of glutamate, glycine and cysteine, is a cysteine-containing tripeptide and is regarded as the most significant cellular redox molecule and acts by scavenging ROS molecules [52]. Specifically, lipid peroxides and hydrogen peroxides produced by ischemic injury are eliminated by glutathione and it functions as a significant antioxidant element in this setting. There is significant evidence that brain damage, such as traumatic brain injury, seizure, ischemia, or hypoglycemia lead to a reduction of glutathione levels [53,54]. Therefore, we investigated whether administration of PCA can restore glutathione levels in hippocampal neurons after global cerebral ischemia. In the present study, we discovered that PCA prevents ischemia-induced glutathione (GSH) decrease in the CA1 region. Therefore, restoration of glutathione levels via PCA administration may exhibit neuroprotective effects. However, it is uncertain how PCA inhibits glutathione reduction after ischemic injury. This question will be investigated in future studies.

**Figure 9:**
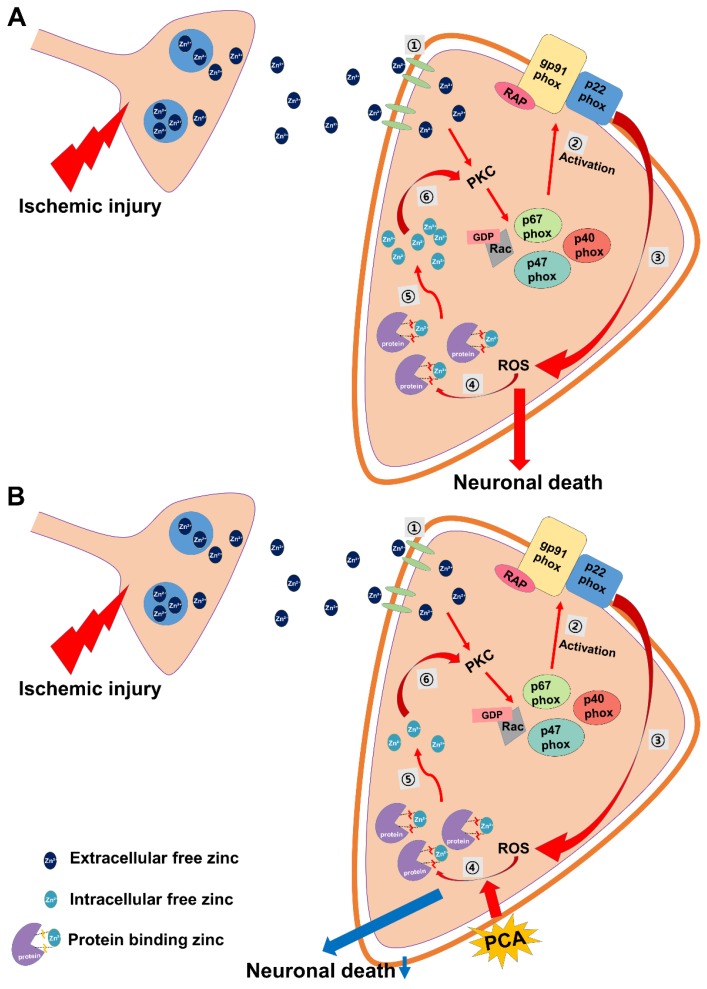
Possible association of zinc, PCA and neuronal death under ischemic conditions. This schematic illustration demonstrates neuronal death by ischemic-induced zinc release. (**A**) (1) Zinc is released into the intracellular space through zinc receptors. (2) PKC and p47 activation and NADPH oxidase activation. (3) Increased ROS formation. (4) ROS inhibits protein zinc binding. (5) Increase of intracellular free zinc. (6) Exacerbated neuronal death by increased intracellular free zinc. When these conditions dominate neuronal death is more likely to occur. (**B**) However, administration of PCA can reduce the intracellular free zinc level by scavenging ROS and thus decrease ischemia-induced neuronal death.

Zinc is well known as an important trace element because of its structural and catalytic functions. Thus, under physiological conditions intracellular zinc levels are very tightly controlled by zinc transporters, zinc binding molecules, and zinc sensors. However, under pathologic conditions such as ischemia, traumatic brain injury or seizure, zinc homeostasis is destroyed. Impairment of zinc homeostasis leads to cognitive impairment and neuronal cell death. In neurons, if there is brain damage, synaptically-released zinc can migrate to neighboring cells [55]. Zinc can directly regulate various intracellular signaling pathways by interacting with protein kinase, receptors, and transcription factors [56]. Moreover, previous studies have suggested that zinc may act as an intracellular signaling molecule or a second messenger in these cells [57,58]. Released zinc stimulates protein kinase C (PKC), p47, NADPH oxidases, ROS production [59]. Zinc-induced ROS formation destroys the sequestration of zinc by zinc-binding proteins, and thus free zinc levels are significantly increased in the intracellular space. Therefore, we evaluated the intracellular free zinc levels with TSQ staining and found that PCA reduced free zinc by scavenging ROS (Figure 9).

Finally, we conducted the adhesive removal test (tape removal test) in order to confirm whether administration of PCA can improve cognitive and sensory function. In our study, we observed that PCA improved ischemia-induced cognitive impairment. Therefore, the present study suggests that PCA administration may be a promising therapeutic tool for reducing hippocampal neuronal death after stroke.

## 4. Materials and Methods

### 4.1. Ethics Statement

The present study is conducted in accordance with the manuals in the Guide for the Use and Care of Laboratory Animals, approved by the National Institutes of Health. Animal studies were in accordance with the stipulation offered by the Committee on Animal Use for Study and Education at Hallym University (Protocol number Hallym-2016-66). We sacrificed animals under isoflurane anesthesia and all procedures were executed to minimize suffering.

### 4.2. Experimental Animals

This study used eight-week old adult male Sprague-Dawley rats (280–330 g, DBL Co., Chungcheongbuk-do, Eumseong-gun, Korea). Animals were maintained one per cage under conditions of constant room temperature (20 ± 2 °C) and humidity (55 ± 5%). Room lights were controlled automatically, turned on and off in a 12 h cycle (on at 06:00 and off at 18:00).

### 4.3. Global Cerebral Ischemia Surgery

Transient global cerebral ischemia was produced under the method introduced by Smith et al. [60]. Rats weighing 280–330 g were anesthetized with 2–3% isoflurane. The body temperature was kept at 37 ± 1.0 °C using a heating pad and heating machine is regulated by a rectal thermistor. A catheter was inserted into the femoral artery to monitor blood pressure and to collect blood samples. We revealed and clamped both common carotid arteries, and the systemic average arterial pressure (MAP) was diminished to 40 ± 5 mmHg by extracting blood (7–10 mL) from the femoral artery into a heparinized syringe for 7 min while maintaining temperature at 37 °C. Successful induction of global brain ischemia was checked by the monitoring of iso-electricity on an electroencephalograph (EEG) (BIOPAC system Inc., Santa Barbara, CA, USA) [27,61]. To monitor EEG patterns, we made bilateral holes in the temporal areas of the skull and then inserted the EEG probes beneath the dura and a reference needle was placed in the neck muscle. The onset of iso-electricity was defined as the point of the last three cortical bursts within a 60 s interval. We conducted reperfusion of blood that was recovered by unclamping both common carotid arteries and reinfusing the extracted blood through the femoral artery after seven minutes of the isoelectric EEG. After reperfusion, and confirmation by the restoration of the baseline EEG signal, rats were given an oral injection of PCA (30 mg/kg, p.o) at recovery of ischemia state. Controls were given 1 mL of 0.9% saline only and the sham operation groups (Vehicle, PCA) (*n* = 5) also received 1 mL of 0.9% saline and PCA (30 mg/kg, p.o). After recuperation from global ischemia, the rats moved their body to the temperature-controlled recovery room and ate a Purina diet (Purina, Gyeonggi, Korea) normally. With severe brain injury, there is the uncommon possibility of neuronal death or seizure in the post-ischemic condition; any animals showing seizure were ruled out from these data analyses. Twelve animals per ischemia group were used in this study. In the entire experiment, the mortality rate was 16.6% and the excluding rate due to seizure was 8.3% (less than 10% after ischemia).

### 4.4. PCA (Protocatechuic Acid) Administration

To confirm the effect of PCA (protocatechuic acid) on neuronal death after global ischemia, the experimental group was divided into four groups (Sham (Vehicle, PCA) and global ischemia (Vehicle, PCA)). The control group was given 0.9% normal saline instead of PCA. After ischemia, animals were injected with PCA (30 mg/kg, p.o.) once a day for one week. All groups were sacrificed at one week after ischemia.

### 4.5. Brain Sample Procedure

Animals were sacrificed at one week after global ischemia, using urethane (1.5 g/kg, i.p.) in order to deeply anesthetize animals. Brains were perfused transcardially with 0.9% saline, and then by 4% paraformaldehyde. Brains were separated after perfusion and then post-fixed in the identical fixative for one hour. The brain samples were cryoprotected by 30% sucrose solution for overnight. When the brain completely sank to the bottom of sucrose solution, we froze and cut whole brains with cryostats at 30 μm thicknesses and kept them in the storage solution until histological evaluation.

### 4.6. Analysis of Hippocampal Neuronal Death

To investigate degenerating neurons after ischemia, brain sections (30 µM) were laid on gelatin-coated slides to stain (Fisher Scientific, Pittsburgh, PA, USA). Fluoro-Jade B staining was performed as depicted by Hopkins and Schmued [62]. Firstly, the slides were put into a 100% alcohol solution for three minutes, 70% alcohol solution for one minute, distilled water, and then into 0.06% potassium permanganate for 15 min. Secondly, the slides were put into 0.001% Fluoro-Jade B (Histo-Chem Inc., Jefferson, AR, USA) solution for 30 min and then washed three times for 10 min in distilled water. We dried brain sample, covered with a cover slide and checked under a fluorescence microscope via blue (450–490 nm) excitation light. To quantify neuronal death, we choose five coronal brain parts from 4.0 mm posterior to the bragma; five brain sections from each animal were then used for analysis. These samples were concealed with encoding of their groups and then offered to a second, blinded tester. A blind tester counted the number of degenerating neurons in constant area (magnification = 10×) of the hippocampal subiculum (900 × 1200 µm), CA1 (900 × 400 µm), CA3 (900 × 1200 µm) and dentate gyrus (900 × 1200 µm) from both hemispheres. The total average number of degenerating neurons from each region was used for statistical evaluation.

### 4.7. Analysis of Live Neurons

To confirm live neurons, we conducted NeuN staining with monoclonal anti-NeuN, clone A60 antibody (diluted 1:500, EMD Millipore, Billerica, MA, USA). It was used as the primary antibody in PBS with 0.3% Triton X-100 overnight at 4 °C. The brain sections were washed three times for 10 min with PBS, and then incubated in biotinylated anti-mouse IgG secondary antibody (diluted 1:250) for two hours at room temperature (Vector Laboratories, Burlingame, CA, USA) and then were washed again Next, the sections were put into the ABC solution (Burlingame, Vector, CA, USA) for two hours at RT on the shaker. Afterwards, the tissues were washed repeatedly three times for 10 min. The immune responses occurred with 3,3′-diaminobenzidine (0.06% DAB agar, Sigma-Aldrich Co., St. Louis, MO, USA) in 0.01 M PBS buffer (100 mL) and 30% H_2_O_2_ (50 µL) for 1 min. We put the brain samples on the gelatin-coated slides. We analyzed the immunoreactions by using an Axioscope microscope [63]. A blind tester counted the number of live neurons in a constant area (magnification = 10×) of the hippocampal subiculum (900 × 1200 µm), CA1 (900 × 400 µm), CA3 (900 × 1200 µm), and dentate gyrus (900 × 1200 µm) from both hemispheres.

### 4.8. Analysis of Oxidative Stress

To evaluate level of oxidative stress in the hippocampus, we conducted immunofluorescence staining with paraformaldehyde-fixed brain tissue. Oxidative stress was detected by measuring the presence of the lipid peroxidation product, 4HNE (4-hydroxy-2-nonenal). Immunohistochemistry with 4HNE (Alpha Diagnostic Intl. Inc., San Antonio, TX, USA) antibodies was conducted according as previously described manual [64]. Brain sections were immersed in a polyclonal rabbit anti-4HNE serum (diluted 1:500, Alpha Diagnostic Intl. Inc., San Antonio, TX, USA) with the PBS containing 0.3% TritonX-100 for overnight in a 4 °C incubator. After we washed the sections three times for 10 min with PBS, these sections were also immersed in a solution of Alexa Fluor 594-conjugated goat anti-rabbit IgG secondary antibody (diluted 1:250, Invitrogen, Grand Island, NY, USA) for two hours at RT. The sections were laid on gelatin-coated slides in order to observe under a microscope. To measure the oxidative injury, we used Image J (v. 1.47c.) program and measured the mean gray value [63].

### 4.9. Analysis of Blood–Brain Barrier Disruption

We measured the degree of leakage of endogenous serum IgG after ischemia in order to investigate the effects of PCA [65,66]. As previously explained, we perfused whole brains. By using fixed brain samples (30 μm thicknesses), we conducted IgG staining with anti-rat IgG (diluted 1:250, Burlingame, Vector, CA, USA), which represents a level of IgG leakage caused by BBB breakdown. The sections were washed as previously described and put into ABC solution (Burlingame, Vector, CA, USA) for two hours at RT on the shaker. The immune responses occurred by 3,3′-diaminobenzidine (DAB in 0.1 M PBS buffer and 0.015% H_2_O_2_). The immune responses were observed through an Axioscope microscope and the whole brain was quantified for the degree of endogenous serum IgG infiltration by using Image J software. The sequence of use is as follows: First, the image is loaded into Image J and the following sequence is applied: Click Image → Adjust → color threshold. Check the threshold color is black and remove the check in the blank of dark background. Then, adjust the brightness and saturation with the same value (the researcher can set this to a value useful to their dataset, but the same value should be used in all images being analyzed from an experiment). Click the menu option Image → Type → 8 bits and then, edit → invert. To measure the area, the menu option Analyze → Measure was selected. We use mean gray value (magnification = 4×).

### 4.10. Analysis of Microglia and Astrocytes Activation

To investigate microglial activation and activated astrocytes, we conducted CD11b and GFAP staining. After washing in PBS, staining was conducted with a mixture of mouse antibody to rat CD11b (diluted 1:500, Serotec) and of goat antibody to rat GFAP (diluted 1:1000, Abcam). Following incubation in PBS containing 0.3% TritonX-100, we left it overnight in a 4 °C incubator. After rinsing, the sections were submerged into secondary antibody (Alexa Fluor 488-conjugated donkey anti-mouse IgG secondary antibody, Alexa Fluor 594-conjugated donkey anti-goat IgG secondary antibody respectively, both diluted 1:250, Molecular Probes, Invitrogen) for two hours at RT. A randomly blinded researcher then measured the intensity of astrocytes in the CA1 region. In the case of microglia, five sections from each brain were scored with same area (magnification = 20×) of the hippocampal CA1 region. Functional standards of microglial activation were their morphology, the number, and intensity of microglial cells. Morphology score of 0: no activated morphology (amoeboid morphology with enlarged soma and thickened processes); 1: 1–45% of microglia; 2: 45–90% of microglia; and 3: >90% of microglia with the activated morphology. CD11b-immunoreactive cell score of 0: no cells are present; 1: 1–9 cells; 2: 10–20 cells; and 3: >20 cells with continuous processes per 100 µm². Intensity score of 0: no expression; 1: weak expression; 2: average expression; and 3: intense expression. Therefore, the total score sums up the three scores (microglial morphology, cell number, and intensity) depending on the categories, ranging from zero to nine (magnification = 20×) [67,68].

### 4.11. Analysis of GSH Levels

To measure glutathione levels, we conducted GS-NEM staining and incubated brain samples with a solution including 10 mM N-ethylmaleimide (NEM, Sigma-Aldrich, St. Louis, MO, USA) for 4 h in a 4 °C incubator. After rinsing in PBS, we stained samples in order to measure glutathione production levels with the primary GS-NEM antibody (diluted 1:100, GS-NEM, Millipore). After this, it was put into a secondary antibody (Alexa Fluor 488-conjugated goat anti-mouse IgG, diluted 1:400, Molecular Probes, Invitrogen) for two hours at RT in darkness. Then we measured GSH levels of selected sections of hippocampus using Image-J software. To measure the concentration of glutathione, we loaded the image into Image J (v. 1.47c) and the following steps were executed: select 5 cells in one brain tissue and then click the menu option Analyze → Measurement. This produced the mean value (generally, in sham group, glutathione was at maximal cellular levels. However, in the ischemia-induced group, glutathione was reduced in degenerating or degenerated neuronal cells) (magnification = 80×) (NCBI, Bethesda, MD, USA).

### 4.12. Analysis of Intracellular Free Zinc Level

Intracellular free zinc was detected using *N*-(6-methoxy-8-quinolyl)-*para*-toluene sulfonamide (TSQ) staining [69]. Rats were euthanized at 3 days after PCA (30 mg/kg, p.o) administration and then the fresh frozen (but not fixed) brains were coronally sectioned at 10 μm thickness in a −15 °C cryostat, then put on gelatin-coated slides and dried. Five evenly spaced sections were selected in the hippocampal region from each brain and dipped in a solution of 4.5 mmol/L TSQ (Enzo Life Science, Enzo Biochem, Inc, Farmingdale, New York, USA, ENZ-52153) for 1 min, then washed for 1 min in 0.9% saline. We observed and photographed each sample with a microscope under 360 nm UV light and 500 nm long-pass filter. To measure zinc intensity, we used the Image J (v. 1.47c.) program and measured the mean gray value.

### 4.13. Behavioral Test

*Adhesive removal test.* To test whether oral treatment of PCA showed improvement of cognitive function after ischemia, we conducted an adhesive removal test for six days from day after the surgery. After an initial adjustment time in a transparent test box (45 × 35 × 20 cm), the rat was lightly restrained to allow adherence of 1 cm square stickers to the palm of each forepaw. And then, we moved the rat quickly into the test box and measured the time to remove the tape. Five trials were undertaken in each test session, with a break time for at least 1 min between trials. A maximum time of 120 s was assigned for each trial. If the stickers could not be removed within this period, a maximum time was recorded.

### 4.14. Data Analysis

Comparisons among experimental groups were executed using analysis of variance (ANOVA) in accordance with the Bonferroni post hoc test. Data were displayed as the mean ± S.D., and differences were regarded significant at *p* < 0.05.

## 5. Conclusions

In the present study, we studied whether PCA known as antioxidant has neuroprotective effects on global ischemia neuronal death. To sum up, we found the following: (1) Administration of PCA decreased degenerating neuron in the hippocampus after ischemic injury; (2) Administration of PCA reduced ischemia-induced oxidative stress; (3) Administration of PCA prevented ischemia-induced blood-brain barrier disruption; (4) Administration of PCA lessened ischemia-induced microglial activation and activated astrocytes in the hippocampus; (5) Administration of PCA prevented ischemia-induced reduction of GSH concentration in the hippocampal neurons; (6) Administration of PCA reduced the intracellular free zinc level in the CA1 region; (7) Administration of PCA improved cognitive and sensory function after ischemia.

Taken together, our present study proposes that PCA has the strongly antioxidant and neuroprotective effects and thus we suggest that PCA can be therapeutic treatment for global ischemia- induced neuronal death.
